# Near-Field Communication in Biomedical Applications

**DOI:** 10.3390/s21030703

**Published:** 2021-01-20

**Authors:** Sung-Gu Kang, Min-Su Song, Joon-Woo Kim, Jung Woo Lee, Jeonghyun Kim

**Affiliations:** 1 Department of Electronic Convergence Engineering, Kwangwoon University, Seoul 01897, Korea; tjdrn6985@naver.com (S.-G.K.); songs922@naver.com (M.-S.S.); wnsdn9504@gmail.com (J.-W.K.); 2 School of Materials Science and Engineering, Pusan National University, 2 Busandaehak-ro 63beon-gil, Geumjeong-gu, Busan 46241, Korea

**Keywords:** near-field communication (NFC), biomedical, applications, sensors, battery-free

## Abstract

Near-field communication (NFC) is a low-power wireless communication technology used in contemporary daily life. This technology contributes not only to user identification and payment methods, but also to various biomedical fields such as healthcare and disease monitoring. This paper focuses on biomedical applications among the diverse applications of NFC. It addresses the benefits of combining traditional and new sensors (temperature, pressure, electrophysiology, blood flow, sweat, etc.) with NFC technology. Specifically, this report describes how NFC technology, which is simply applied in everyday life, can be combined with sensors to present vision and opportunities to modern people.

## 1. Introduction

Near-field communication (NFC) is a type of radio frequency identification (RFID), which is a contactless communication technology that operates in a frequency range centered on 13.56 MHz. This technology allows users to send identification information wirelessly to the receiver for tracking and security purposes. NFC technology is only usable within a limited distance; therefore, it is relatively secure and is used in various areas, such as industry [1,2,3], medicine [4,5,6,7,8,9,10,11,12,13], and banking and finance [14,15,16]. Most electronics, such as modern smartphones and credit cards, are equipped with basic NFC technology, and are naturally used in everyday life [17,18]. In addition, modern NFC technology has been combined with the internet of things (IoT) to control products [19] and identify various objects (clothes, textile, fruits, etc.) with tags [20,21,22,23].

Public interest in biomedical applications with NFC technology has increased significantly [24,25]. There have been attempts to continue to increase the distance over which NFC technology can be used to exchange data and transmit and receive power [26,27]. By increasing the usable distance, the spatial limitations of conventional NFC can be dramatically expanded. Accordingly, the potential application of NFC technology in the biomedical field is emerging as a topic of interest [8,9,10,11,12,13]. In this field, the focus is on the advantages of the integrated application of NFC technology and sensors supporting battery-free usage. The advantage of a battery-free power supply method is that parameters such as the device weight, volume, and thickness can be dramatically reduced. Due to its ease of miniaturization, NFC technology has been optimized for integration with wearable sensors. NFC technology has a critical advantage, in that it enables real-time biosignal monitoring in a “non-cognitive” state anywhere in the daily life of the user. Bluetooth and WiFi require batteries for communication, whereas NFC technology does not require complex processes such as pairing and transmission control protocol connections [28,29].

This review introduces the history of NFC technology, including the design, materials, and fabrication techniques of NFC antennas which determine the performance of NFC devices. It further describes the existing application methods, self-customized health monitoring, and applications for drug identification and disease diagnosis in hospitals, and lists the purposes and characteristics of such devices. Then, it discusses the standards integrated with the NFC operating mode and proposes the parameters and design logic of a loop coil antenna for data communication and power transmission and reception. In NFC, an analog voltage output is provided by the reader from the magnetic field of the receiver, created by the inductive coupling between antennas, and powers the various sensors and integrated circuits. Parameters such as the quality factor (Q) and read range of an electronic device that determine the performance of an NFC device depend on the antenna material, design, and manufacturing technology [30,31].

The rest of this paper is organized as follows. Section 2 introduces the existing research on systems that combine NFC and biosignal sensors in biomedical applications. Section 3 presents the trends, applications, and development possibilities for temperature and pressure sensors, electrophysiology sensors, blood flow sensors, and sweat sensors combined with NFC technology. It describes systematic and theoretical studies conducted using radio frequency (RF) readers that generate data and power transmission through resonant inductive coupling using the NFC protocol to support stable device operation under various practical conditions. Furthermore, it discusses the use of the previously mentioned sensors in experimental studies to accurately measure the parameters of subjects with healthy skin and various skin pathologies. These sensors using NFC will have implications for various aspects of human life, including skincare, clinical health, and athletic performance. Finally, Section 4 provides the conclusions regarding the benefits of this technology and its application scope.

## 2. Near-Field Communication

### 2.1. History of NFC

NFC technology was developed by Thomas Edison in a radio frequency experiment conducted in the late 1800s. NFC, first patented by Charles Walton in 1983, is rooted in RFID. In 2002, Sony and NXP Semiconductors collaborated to develop a new type of NFC technology. In 2004, an official NFC forum was formed, and Sony, Philips, and Nokia focused on combining NFC with production. In 2006, the first phone with NFC was launched by Nokia, representing the use of NFC as a way to share information rather than simply as a payment method. In 2009, the NFC Forum established peer-to-peer (P2P) standards to enable data transmission and reception between phones with NFC. From 2010 to 2016, various applications such as Android phones with NFC, smart tags, secure transactions, and Felica cards were developed. By 2020, NFC was being utilized in various sensors as well as in IoT, medical, and home applications [32].

In traditional applications, NFC enables users to pay by smartphone without a credit card, smart card, or cash [17,18]. NFC is also used as an authentication method to control access rights by storing the biometric information of users [6,7,33]. In addition, it is used to check the attendance of students and office workers, store coupons, and mobile ticketing. As such, traditional NFC has been utilized for payment, personal authentication, and commercial convenience. This paper describes how the applications of NFC can be expanded to everyday life.

In daily life today, an NFC reader can simply be connected to an NFC tag device to identify the family members stored in the back-end system and monitor individual health information [7,34,35]. For many years, healthcare and wellness information systems have attracted research interest. In particular, many studies on personalized healthcare for individuals at home have been conducted. Most home health monitoring systems track health or lifestyle information, some of which send health information to the hospital. Biosignals are among the most important indicators needed for disease prevention, especially in infants and seniors [36,37].

All patients in hospitals have different diseases and symptoms. When a doctor operates on a patient, there is a possibility of confusion about the disease and treatment of the patient, which can be detrimental to the treatment or even lead to death. Using NFC to create powerful medical systems can protect patients from fatal medical mistakes. To reduce diagnostic errors, the NFC tag is used to identify a given drug [38,39]. Various NFC sensors such as heart monitors, temperature sensors, and blood pressure sensors not only collect real-time biosignal data from patients, but also send them to homes and hospitals to help track patient information. In addition, contemporary technological developments have helped support people with visual impairments in their daily work and enabled them to overcome various problems in their daily lives. In many countries, NFC tagging technology is used to develop assistive tools for people with visual impairments in various low-cost applications to overcome the problem of assistive tools [40,41].

### 2.2. NFC Circuit and Design

NFC offers three different operation modes: reader/writer mode, P2P mode, and card emulation mode. Reader/writer mode supports one-way communication. In this mode, data are transferred from the NFC tag to the NFC device or from the NFC device to the NFC tag. P2P mode supports two-way communication, thus data are transferred between two NFC devices. In card emulation mode, data are transferred from the NFC device to the reader. NFC enables both convenient and intuitive interaction. A master device with information can be read by other passive mode devices, whereas a passive mode device, conversely, cannot transmit information to the master device. Devices in active mode can collect or change information from tags in passive mode [32].

NFC is defined in the standards ECMA-340 and ISO/IEC 18092. NFC incorporates various existing standards, such as ISO/IEC 14443 Type A and Type B and Felica. Among the NFC standards, ISO/IEC 14493 Type B and 15693 support active–passive communication by supporting passive mode. On the other hand, ISO/IEC 18092 supports active–active communication with the addition of P2P technology [32].

The optimal size of the loop coil used as the antenna is determined by its inductance, Q factor, and read range. In an NFC system, the resonant frequency of the device should be close to 13.56 MHz. The antenna input inductance is calculated based on the parameters of the loop coil [30,31,32]. For example, an antenna based on planar square coils was reported which consisted of three turns of 30 mm × 30 mm, with an access line of 5 mm to connect the inner and outer paths of the square coil. The simulated antenna input impedance indicated a reactance of j40 Ω and a very low-input resistance at the operation frequency [42]. Another report described research on a stretchable coil that had overall dimensions of 74 mm × 50 mm with six turns, a ribbon width of 400 μm, a ribbon gap of 400 μm, and a ribbon thickness of 18 μm. In this work, the inductance was estimated to be about 4.5 μH. To tune the resonance frequency further, an external capacitor was placed in parallel to the internal resonance capacitor [43].

**Figure 1:**
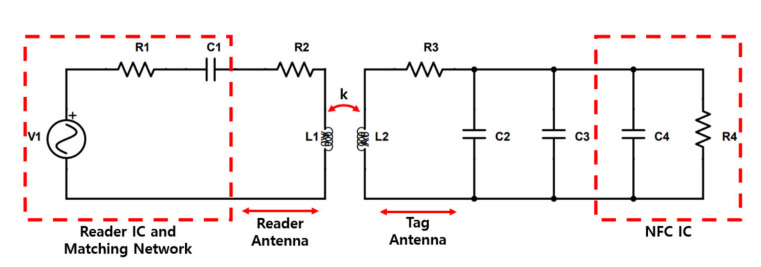
Wireless power transfer circuitry between the reader antenna and tag antenna including reader integrated circuit (IC), matching network, and near field communication (NFC) IC [44].

Figure 1 is an equivalent circuit model of an NFC system, consisting of a transmitter and a receiver. The reader integrated circuit (IC) and matching network parts have been simplified for easy viewing of the RF front-end. The total input impedance, denoted by R_1_ and C_1_, is an important parameter of the matching network that determines whether or not to transmit maximum power. The tag antenna consists of an inductance L_2_, an equivalent resistance R3 considering the power loss of the antenna, a parasitic capacitance C_2_ for interconnection with the antenna, and a tuning capacitance C_3_ for regulating the resonant frequency.

The loaded Q factor of the tag (*Q*_2*L*_) is given by the hyperbolic average of the Q factor of the tag’s antenna (*Q*_2_) and the loaded Q factor of the IC (*Q_L_*):

(1) $$Q_{2L}=\frac{1}{\frac{1}{Q_{2}}+\frac{1}{Q_{L}}}\approx Q_{L}$$

(2) $$Q_{L}=\frac{R_{4}}{\omega L_{2}}$$

A tuning capacitance is added to the internal capacitance of the NFC IC (20–50 pF) to adjust the resonance at 13.56 MHz [44]:

(3) $$f_{r}\approx \frac{1}{2\pi \sqrt{L_{2}\left(C_{2}+C_{3}+C_{4}\right)}}$$

## 3. Biomedical Applications

### 3.1. Temperature and Pressure Sensors

**Figure 2:**
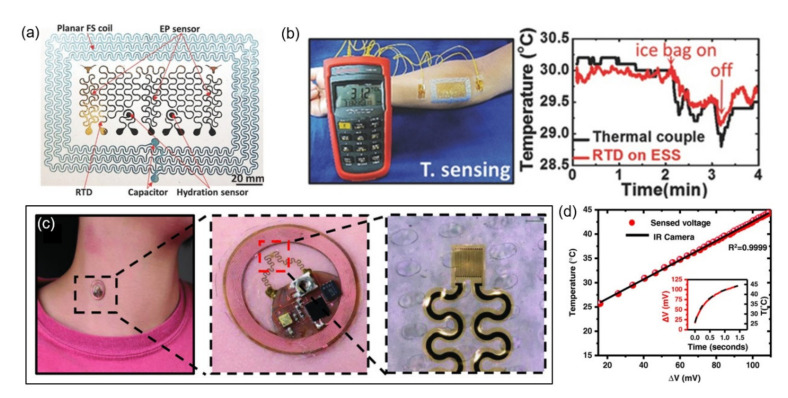
Examples of NFC applications for temperature and pressure sensing. (**a**) Construction of a multifunctional epidermal NFC sensor. FS, filamentary serpentine; EP, electrophysiological; RTD, resistance temperature detector [45]. (**b**) Temperature sensing results [45]. (**c**) Photographs of a NFC temperature sensor on a neck [46]. (**d**) The result of converting the voltage measured wirelessly to temperature through calibration of the infrared (IR) camera [46].

In biomedical applications, the most commonly accessed types of information are temperature and pressure. The epidermal sensor in Figure 2a,b proposed in this paper is a preliminary study before it is combined with NFC technology [43,45]. The device completed by developing the previous research is advantageous in introducing the ultra-thin form because it is powered by a wireless power supply method and does not require a battery. Ultra-thin and ultra-soft epidermal biometric temperature sensors combined with NFC technology will be introduced here. These sensors can be layered on any part of human skin like temporary tattoos. They can also fully comply with the microscopic shape of human skin, resulting in a low electrode-to-skin interface impedance and high signal-to-noise ratio (SNR). Wireless power supply through inductive antennas has been presented using commercially available ultra-low power NFC chips that can transmit power and data wirelessly. Therefore, an e-tattoo with an integrated NFC chip and antenna enables wireless biometric identification without a battery. Using wireless communication techniques means addressing the shortcomings of high power consumption. In this device, the power consumption of the NFC chip (RF430FRL152H, Texas Instruments Inc., Dallas, TX, USA) in standby mode is very small, about 24 μW. This NFC power supply system provides 1.5 mW of rectified output power to operate an external thermistor (NTCG164KF104F, TDK Corp., Tokyo, Japan) and a phototransistor (TEMT6200FX0l, Vishay Intertechnology Inc., Malvern, PA, USA). When the thermistor is activated, it has a low reference impedance, allowing a low current of 2.4 μA to flow, and the device’s self-heating is minimized [43]. Figure 2a proposes an integrated device in which impedance sensors, electrodes, and stretchable coil are combined. Figure 2b depicts the photos of the device attached to the forearm, and the body temperature data compared to the commercial product. Simultaneous measurement with a commercial thermometer (TMD-56, Amprobe Instrument Corp., Everett, WA, USA) proved the performance of a soft and flexible device.

The thermal sensor can be attached to almost any part of the body, including the nails, and comes with a sensor that measures the body temperature through the blood flow. The skin-like thermal sensor combined with the optimized platform is battery-free and can be used with smartphones. In this platform, data and power transfer occurs via a resonant inductive coupling to the RF reader and NFC protocol. The signal amplified by the circuit consisting of analog drivers and amplifiers is received by a 10-bit analog-to-digital converter (ADC) with a full range of 300 mV (300–600 mV) of the NFC chip (SL13A, ams AG, Unterpremstätten, Styria, Austria). As a result, the measurement accuracy of around 80 mK was obtained due to software limitations, but the designed analog circuit could achieve a measurement accuracy of around 20 mK. The skin-like thermal sensor can decompose the thermal conductivity of 0.02 Wm^−1^K^−1^, and has a measurement uncertainty of 5 to 10%. The persistence and stability tests of the NFC temperature sensor conducted on human subjects under various conditions ensure the reliability of the operation of the device [46]. Figure 2c shows an optical image of an epidermal wireless thermal sensor attached to the skin of the neck. The platform consists of two mechanically distinct components. The first component is assembled on a flexible printed circuit board (flex-PCB; flexural rigidity, EI ≈ 3000 Pa m^3^) by integrating an induction coil for wireless power harvesting and an integrated circuit for NFC-based data transmission and analog signal conditioning. The second component is a pattern designed by the photolithography of Cr/Au (10/100 nm) encapsulated with a thin (3 µm) polyimide layer on an elastomeric substrate (80 µm, EI ≈ 0.3 Pa m^3^). Figure 2d depicts the result of converting the voltage change measured wirelessly to temperature.

**Figure 3:**
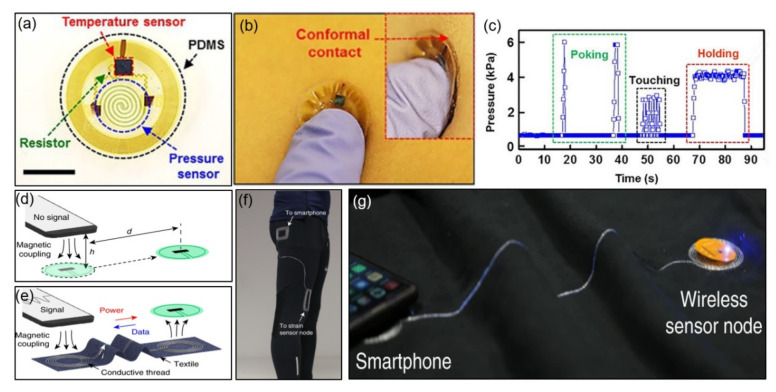
Examples of NFC applications for temperature and pressure sensing. (**a**) Temperature and pressure sensor integrated with an NFC chip [47]. (**b**) Photograph of an NFC sensor pressed with the fingertip [47]. (**c**) Pressure measured by a device on the left forearm [47]. (**d**–**g**) Photographs of NFC-enabled clothing [48].

The epidermal NFC temperature and pressure sensors have been proposed that can be used throughout the body. These sensors map the skin temperature and pressure in specific areas of the body to easily identify human health and provide predictive information for disease prevention. Here, the helical structure, consisting of a thin single-crystal film of silicon, acts like a pressure-sensitive element through its piezoresistive properties, and its resistance changes with mechanical deformation. The spiral shape improves the uniformity of the strain distribution under pressure, compared to a simple linear design, which promotes stable operation on the skin surface. The temperature and pressure sensors used in the study consume relatively low power for operation. It draws a low current of 2 μA at 1.5 V (~3 μW) in standby mode and 150 μA at 1.5 V (~225 μW) in operating mode. The NFC chip (SL13A, ams AG, Unterpremstätten, Styria, Austria) with 10-bit ADC, mentioned in the previous paragraph, minimizes signal interference due to noise and electromagnetic interference by the external environment through the sequential data acquisition method of multiple sensors. The researchers checked full-body coverage by installing two reader antennas on the bed to determine the sensor’s operating range. They completed communication and powering tasks for 65 individual sensors in a time-sequential manner and achieved a reading time of less than three seconds [47]. Figure 3a provides a top view of an NFC microchip, temperature sensor, and silicon membrane pressure sensor coated with polydimethylsiloxane (PDMS). Figure 3b shows an optical image of the device in conformal contact with the skin. Figure 3c illustrates the pressure fluctuations recorded wirelessly while applying various forces with the tip of a finger. A 6 Hz sampling rate and pressure fluctuation results for poking (green), touching (blue), and holding (red) were obtained.

As a further application, near-field-enabled clothing, which requires no battery, is close to functional fiber patterns, and allows wireless power and data transmission from the sensor has been reported. Fabric-based pattern and near-field-enabled clothing integration systems using inexpensive conductive threads and computer-controlled embroidery are free of fragile silicone components. Although current clinical monitoring systems require sensors to be wired to a central hub for power supply and data collection, this form limits physical movement and has limited use outside clinical settings. This system overcomes the limitations of conventional wired monitoring by integrating a near-field-responsive inductor pattern that can wirelessly connect multiple skin-mountable sensors to a reader up to 1 m away. The researchers proposed a textile design and wireless spinal posture monitoring compatible with NFC-enabled smartphones and devices, as well as a way to measure temperature and gait continuously during exercise. They calculated the minimum efficiency required for energy and data transmission as about 2%, considering the power consumption of the sensor node of 4 mW and the reader’s output power of 200 mW. The sampling rate for a single sensor was 8 Hz, and the sampling rate for six multiple sensors was reduced to 1.3 Hz, but the total power consumption of the reader was not affected by this because the antenna output power was constant. In a node based on an NFC tag with an integrated negative temperature coefficient of resistance (NTC) thermistor (ERTJ1VS104A, Panasonic Corp., Kadoma, Osaka), the power consumption of the front-end circuit for the temperature sensor was 1.15 μW and the total power consumption was less than 4 mW [48]. Figure 3d,e demonstrate that a time-varying magnetic field (13.56 MHz) induces current throughout the relay to generate magnetism through the close proximity of the wireless reader to the inductor pattern (hub). Figure 3f shows the method of maintaining a connection between the sensor node and reader through near-field relays embroidered on the pants. Figure 3g depicts a wireless power supply at both ends of the terminal and a connection with a sensor outside the NFC range.

There are points to be aware of when combining the temperature sensor and NFC technology. When researchers use the onboard temperature sensor built into the NFC IC tag (SL13A, RF430CL330H, etc.), there is a possibility that self-heating may occur due to the limiter inside the tag. The device’s self-heating due to external power supply affects the accuracy of the temperature measurement, interfering with body temperature data collection. In the stage of designing the reader and tag antenna circuits, post-processing and calibration steps can be eliminated by inserting a structure that dynamically controls the transmit power.

### 3.2. Electrophysiology Sensors

**Figure 4:**
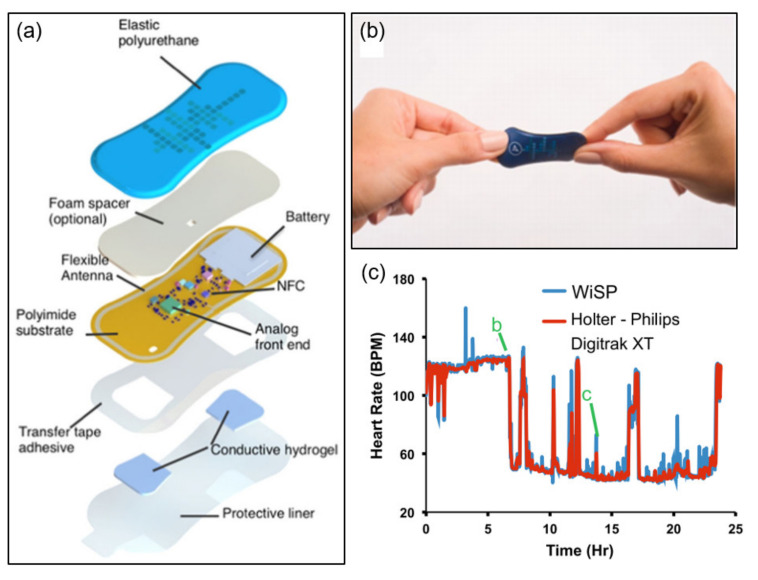
Examples of NFC applications for electrophysiology sensing. (**a**) Construction of a soft flexible cardiac sensor [49]. (**b**) The device twisted and bent [49]. (**c**) Results of comparing the heart rate measured by the sensor with commercial products [49].

Traditional heart rate monitoring devices use optical and electrode-based sensors to measure biosignals over a wire. However, these form factors are not suitable for use in external or home environments. This section describes flexible electrocardiogram (ECG) sensors that can be attached to the epidermis. Furthermore, a highly flexible epidermal ECG and heart rate wearable sensor that emphasizes low cost, lightweight (1.2 g) energy harvesting has been proposed. The sensor’s onboard hardware utilizes instrumentation amplifiers and filters to regulate potentials and signals and reduce common-mode signals. The microcontroller has been designed to coordinate the switching between battery and NFC energy harvesting and to control the surge current when the system is activated, optimizing power consumption [49]. Figure 4a illustrates the main structure of the highly flexible wearable cardiac sensor design, as well as the multilayer conformal mechanism leading to tight skin bonds and strong cardiac signals. This device consisting of multiple polymeric, electronic, adhesive, and hydrogel layers uses two filter settings tailored for ECG and heart rate signal capture. ECG filters can be used to visualize P- and T-waveforms, whereas heartbeat filters that operate in low passbands can eliminate P- and T-waveforms, as well as most motion and muscle activation artifacts. Figure 4b depicts the device in a mechanical twist. Figure 4c shows the results of recorded heart rate data compared to a commercially available device for healthy subjects. Comparison with commercially available products confirmed a 95.4% correlation, and demonstrated that the device (the ultra-low-power biosensing platform introduced in this study) can easily be applied to subjects such as athletes and heart patients.

**Figure 5:**
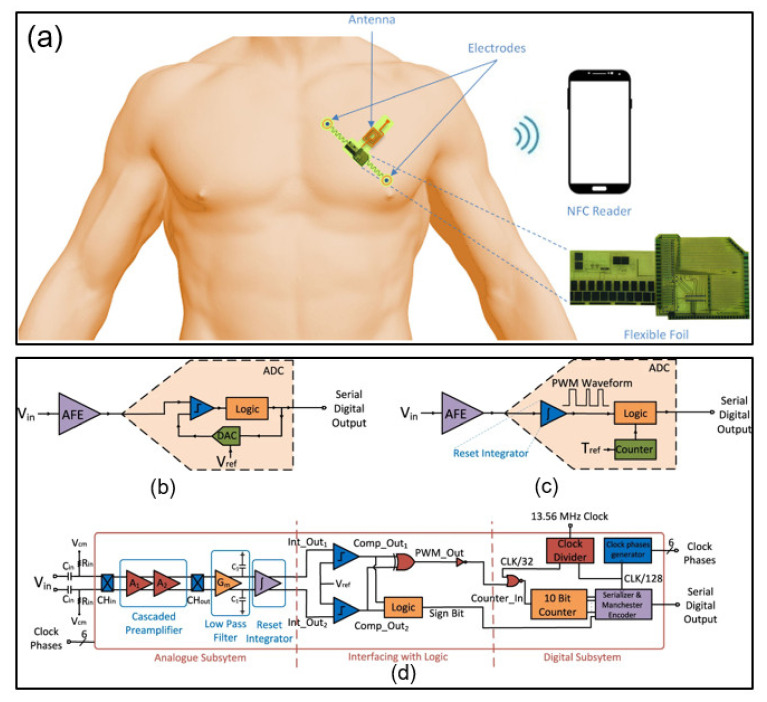
Examples of NFC applications for electrophysiology sensing. (**a**–**d**) Schematics and architecture of an NFC sensor system [50].

Previous research has offered NFC-enabled flexible ECG patches implemented on foil using self-aligned indium–gallium–zinc oxide thin film transistors (IGZO TFTs). The device amplifies the collected ECG signal and converts it into a sequence signal, which highlights the advantage of a state-of-the-art wearable biosignal monitoring ECG sensor based solely on flexible electronics and utilizing TFT-based circuitry. The sensor has no solid active components; therefore, the system is suitable for the human body and convenient to use. In addition, because the entire system is implemented with flexible TFTs, additional processing steps and integration costs are reduced, enabling solutions that are compatible with single use [50]. Figure 5a presents a representative application direction. Figure 5b shows a biopotential acquisition system consisting of an analog front-end (AFE) block and a typical ADC block consisting of a comparator, digital-to-analog converter (DAC), and logic. It is difficult to create a voltage reference because the main components used in the amorphous indium gallium zinc oxide (a-IGZO) cannot be employed in the a-IGZO process with the latest technology. Consequently, the input data are digitized in the time domain in this paper. Figure 5c depicts a simple biopotential acquisition system implemented with a-IGZO, and Figure 5d shows the detailed architecture of the presented system.

### 3.3. Blood Flow Sensors

**Figure 6:**
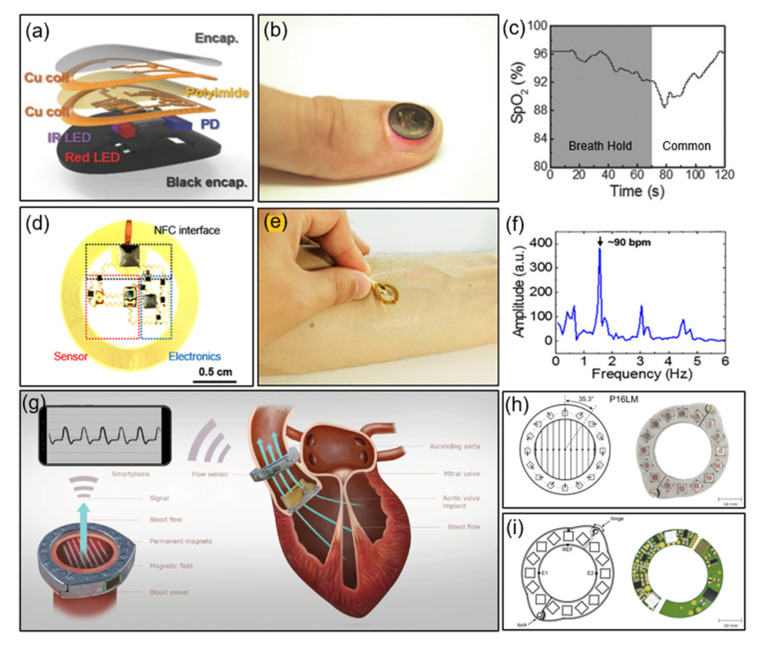
Examples of NFC applications for blood flow sensing. (**a**) Construction of an NFC-enabled pulse oximeter device. PD, photodetector [51]. (**b**) Photograph of an NFC device on a fingernail [51]. (**c**) Results of SpO_2_ during a breath-hold test [51]. (**d**) Top view of an NFC heart rate sensor [52]. (**e**) Photograph of an NFC device on skin [52]. (**f**) Biosignal data measured by the device [52]. (**g**–**i**) Construction of an NFC heart valve monitoring device [53].

This section discusses the materials and device concepts for flexible platforms. Based on an optoelectric measurement method, this ultra-thin blood flow sensor performs photoplethysmogram (PPG) data measurement and transmission. The authors previously presented quantitative results on blood oxygenation, heart rate, and heart rate variability. Here, a thin and flexible micro-functional system capable of attaching to almost any part of the body, including fingernails and toenails, is presented. The electronic system, built around a double-layer loop antenna and a microcontroller to improve inductance and Q factor, makes contact with the user comfortable. As a result, Q ≈ 16 and a relatively low input impedance was achieved by utilizing a double-layer coil and thick, low-resistance Cu trace [51]. As shown in Figure 6a, a multilayer layout using a bilayer loop antenna maximizes the energy harvesting efficiency and wireless data communication distances, and provides compact electrical routing between closely spaced components. In addition, the PDMS encapsulation with black dye directs conformal contact with the toenails or other parts of the body and protects against mechanical damage. Figure 6b provides a photograph of a device that works while attached to a fingernail. Figure 6c presents the experimental results of respiratory discontinuation and breathing through a device attached to the fingertip. By providing a vision for achieving a level of convenience inaccessible to conventional systems, the system can easily be adapted by adding an NFC reader to everyday items. Furthermore, blood oxygenation information can easily be monitored wirelessly without interruption during daily activities, and the millimeter-scale, thin, and lightweight device offers greater freedom of choice in terms of attachment locations.

On the other hand, the heartbeat and time dynamics monitoring of arterial blood flow, tissue oxygenation, ultraviolet (UV) dose measurement, and four-color spectral evaluation of the skin can be performed as follows, using a battery-free, flexible optoelectronic system for wireless optical characterization of the skin. This system utilizes multicolor luminescence and detection to diagnose the optical properties of the skin and peripheral vascular disease. Time-multiplexed amplified and digitized signals monitor the heart rate, tissue oxygenation, pressure pulse dynamics, UV exposure, and skin color through an integrated collection of small light-emitting diodes (LEDs) and photodetectors. AC voltage is applied to the SL13A (ams AG, Unterpremstätten, Styria, Austria) bare die chip supporting 10-bit ADC with rectification and single analog input capability. It has a maximum power of about 12 mW depending on the coupling efficiency [52]. Figure 6d shows an image of a device consisting of an IR LED, a photodetector, an amplifier, a resistor, and an induction coil. Figure 6e shows an image of the device attached to the forearm. A single LED obtains the systolic peak and relaxation notch data, and Figure 6f illustrates the three harmonics obtained through Fourier transform.

Finally, we introduce the characteristics of an implantable magnetic blood flow sensor optimized for small size and low power consumption for battery-free operation. The sensor enables wireless and battery-free blood flow recording using magnetic flow meter technology, and the sensor system monitors the characteristic flow downstream of the valve, facilitating the remote management of patients undergoing bioprosthetic heart procedures. To predict the rate of deterioration and valve endurance of the prosthetic heart valve (BHV), this system can monitor the BHV function with minimal overhead and can be used to automate the data collection process The device operated through inductive coupling with the smartphone’s internal antenna consumed 380 μA of current at a rectified supply voltage of 3.4 V. The current consumed by the NFC chip (SL13A) was 150 μA and the current consumed by the circuit for sensing was 230 μA; therefore, the resulting device’s final power consumption was 1.3 mW. The device with an effective sampling rate of 60.2 Hz presents a form factor optimized for miniaturization of the magnetic ring structure [53]. Figure 6g provides a schematic diagram of an implantable magnetic flow sensor attached to the ascending aorta. It receives measurement data with a smartphone and supplies inductive power to the equipment. Figure 6h,i depict a prototype of a Halbach ring designed using neodymium magnets and 3D printed titanium rings. By bringing the implant antenna near the transmitting coil antenna, the voltage is induced and rectified, providing a rectified voltage of 3.4 V and current of up to 4 mA to operate the integrated circuit.

### 3.4. Sweat Sensors

**Figure 7:**
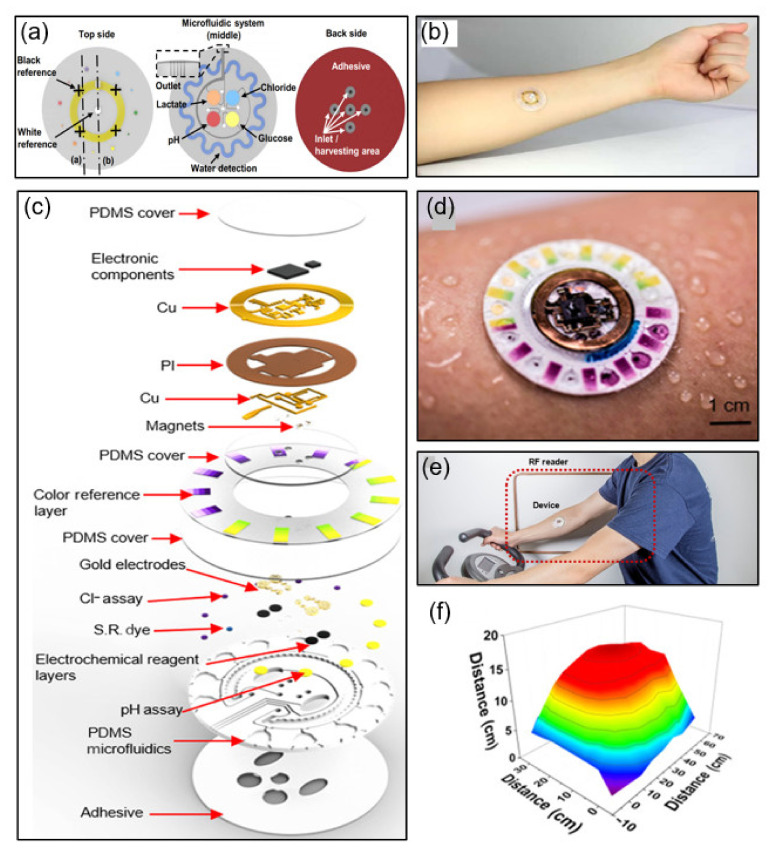
Examples of NFC applications for sweat sensing. (**a**) Construction of an NFC sweat monitoring device [54]. (**b**) Photograph of the sweat monitoring device [54]. (**c**) Construction of a integrated NFC sweat sensor [55]. (**d**,**e**) Photograph of the device attached to the forearm during sweating [55]. (**f**) Results of reading distance between the device and reader antenna [55].

As a sweat sensor, a flexible microfluidic device that sticks to human skin is proposed. It incorporates wireless communications electronics that can tightly and firmly bond to the skin surface without chemical and mechanical irritation. This soft, flexible, and stretchable system allows sweat to initiate routing spontaneously through a set of microfluidic networks and reservoirs. The device can be mounted on multiple parts of the body without chemical and mechanical irritation, including biocompatible adhesives and flexible, stretchable materials, and waterproof interfaces. Colorimetric detection measures the total sweat loss, pH, lactic acid, and chloride and glucose concentrations, and the data are transmitted wirelessly based on an NFC system [54]. Figure 7a shows the integrated system structure diagram divided into the top, middle, and back sides. On the top side, the reference color markers are integrated with the NFC electrode. The middle side consists of an integrated system of microfluidic channels for colorimetric detection. The back side has a uniform layer of adhesive and openings that define sweat access and openings to channels.

One study also proposed a hybrid, battery-free skin mounting system for sweat detection. This completely non-invasive sweat sensor is non-irritating and is designed to replace disposable microfluidic systems by reusing only detachable electronic modules. Modules based on simplified miniaturization and low-cost NFC technology are combined with a disposable microfluidic form factor containing colorimetric reagents. The platform includes an RF system-on-chip consisting of an ISO 15693 compliant 14-bit ADC and an integrated RF front end with a microcontroller. The 1 MΩ load resistor limits the overall voltage error of colorimetric detection experiments to only 2 mV [55]. Figure 7c shows a schematic of a fully hybrid battery-free system. This system includes a silicone elastomer patterned with soft lithography technology, a separate set of chambers for colorimetric and electrochemical sensing, a ratchet channel for quantifying sweat rate and loss, and microchannels of passive capillary burst valves for routing sweat. Figure 7d,e depict a battery-free NFC electronic device attached to an arm.

**Figure 8:**
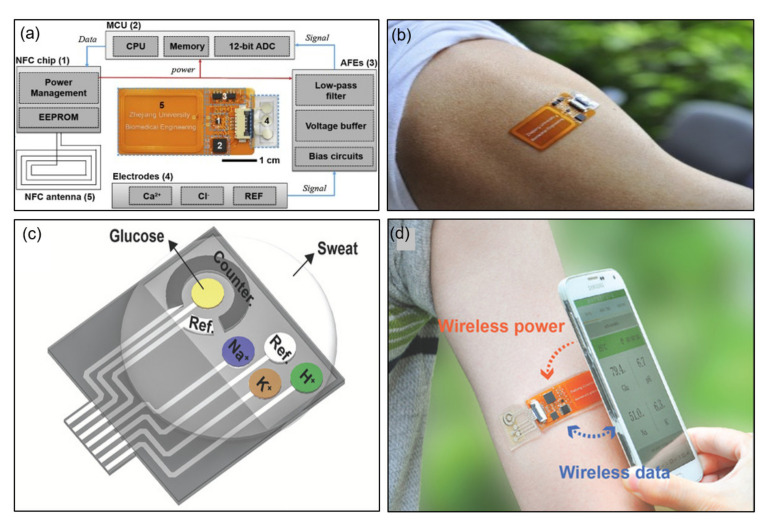
Examples of NFC applications for sweat sensing. (**a**) Block diagram of an electrochemical patch. MCU, microcontroller; EEPROM, electrically erasable programmable read-only memory [56]. (**b**) Photograph of the patch on the arm [56]. (**c**) Construction of a sweat sensor [57]. (**d**) Photograph of the device on the arm [57].

This effective integrated system comes with a fully integrated sensor for analyzing sweat metabolites and a sensor array with NFC technology. Research has been published regarding a sensor array that integrates wireless power harvesting, field signal processing, and wireless data transmission to provide wireless power to patches with NFC-enabled smartphones and to obtain analysis through inductive coupling between antennas. Capable of real-time detection of calcium and chloride ions in various biological fluids, the ion-selective electrodes (ISE) of the sensor are designed to be flexible to accommodate skin modifications [56]. Figure 8a is a block diagram of a smartphone-based sensing system that includes an electrochemical patch and NFC-enabled reader. Inductive coupling for power and data transmission connects the reader and patch. Functionally, the patch consists of an NFC antenna, an NFC chip (NT3H2113N, NXP Semiconductors N.V., Eindhoven, Netherlands), a microcontroller (MCU, MSP430FR2632, Texas Instruments Inc., Dallas, TX, USA), an analog front end (AFEs, based on TLV2401, Texas Instruments Inc., Dallas, TX, USA), and electrodes and is divided into five parts. Figure 8b shows a photorealistic image of the patch attached to an arm. The patch offers the possibility of wireless power and data transmission using NFC technology, overcoming the miniaturization and flexibility limitations of the existing system. Although there are limitations such as short operating distance and power supply constraints, this system is more convenient than the existing system.

Here, a battery-free wireless and epidermal chemistry system is described that employs NFC and printing technology in the design. Without wired connections to conventional batteries or external stations and attaching comfortably to the skin, the system detects glucose, sodium, potassium, and the pH in sweat. The voltage output (≈2.75 V) refined through the power management of the NFC chip enables the operation of the entire circuit of the MCU and AFE [57]. Figure 8c provides a schematic of a four-channel electrode array for detecting glucose, H^+^, Na^+^, and K^+^. By modifying the electrodes with specific surface chemistries, the system performs multiple detections of sweat analytes. Figure 8d depicts a smartphone placed close to the device to power and transfer data to the device when attached to the arm of the user. The multiplexed electrical sensor shows a negligible response to the interfering material and a sensitive response to the target analyte.

### 3.5. Hospital Applications

**Figure 9:**
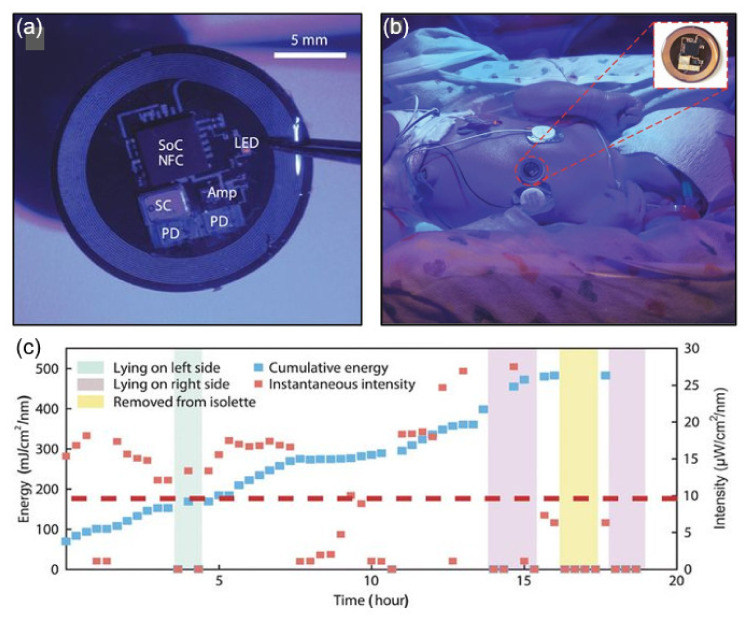
NFC application in hospitals. (**a**) Photograph of a blue light dosimeter/photometer for hospital application. SoC, system on chip; SC, supercapacitor [36]. (**b**) Photograph of the NFC device on the chest of a jaundiced infant [36]. (**c**) Results from the NFC device on the chest of a jaundiced infant [36].

This section presents examples of biomedical applications for millimeter-scale wireless and battery-free NFC platforms with various operating systems. UV radiation from the sun can seriously affect human health, but controlled amounts of electromagnetic radiation can be used positively. Optical metrology, optoelectronic design, and wireless operating mode platforms have been proposed that serve as the basis for miniaturization, low cost, and battery-free devices for precise dose measurements at multiple wavelengths. By utilizing NFC technology, an appropriate antenna layout, and a manufacturing approach for flexible electronics and electronic circuit design, the system enables UVA, UVB, visible, and IR radiation monitoring. The dosimeter for blue light therapy implements continuous wireless data acquisition with a long range reader RF antenna (30 cm × 30 cm, reading range 10–30 cm) placed under the bed [36]. Figure 9a depicts a dosimeter/photometer designed to monitor blue light exposure in a neonatal intensive care unit (NICU), and Figure 9b shows a device attached to the chest of a jaundiced infant receiving phototherapy. The device performs cumulative and instantaneous sensing through ADC1 and ADC2, respectively, on a single NFC chip. The cumulative sensing circuit follows reset detection of the UVA dosimeter and the blue light photodetector (PD) using Reset, MOSFET, and GPIO. The instantaneous sensing circuit couples an amplifier driven by the antenna-rectified voltage (VDDH) to the blue light PD. By powering the sensor with a reader antenna, the digital output signal of the ADC can be transmitted wirelessly over an NFC link. Figure 9c presents instantaneous intensity and dose measurements over time for blue light therapy in jaundiced infants admitted to an NICU.

**Figure 10:**
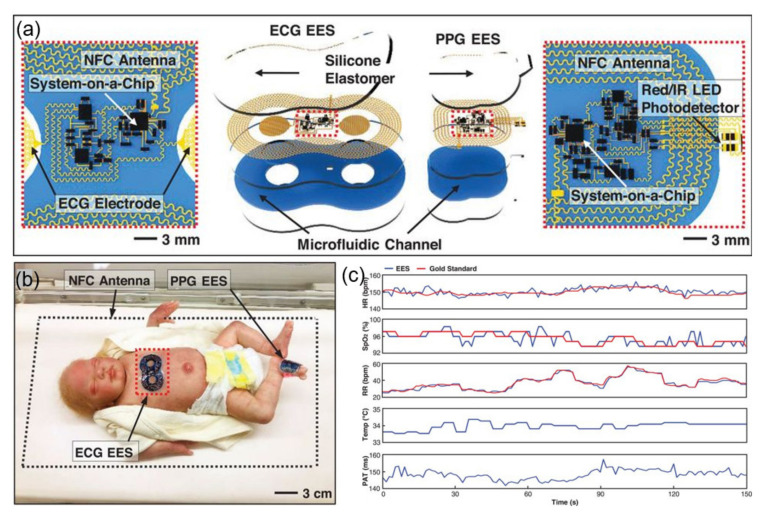
NFC application in hospitals. (**a**) Construction of an NFC electrocardiogram (ECG) sensor. EES, epidermal electronic system; PPGs, photoplethysmograms [37]. (**b**) Photograph of the ECG sensor on the chest and foot of an infant [37]. (**c**) Biosignal results compared to gold-standard monitoring equipment [37].

An ultra-thin skin-like wireless module for complete biosignal monitoring in NICUs also exists. The wireless battery-free module, presented in Section 3.2 as an epithelial electronic system (EES) for biosignal monitoring, performs ECG and PPG data and skin temperature recording. They proposed a solution incorporating a magnetic loop antenna that allows simultaneous wireless data transmission and wireless power supply. It targets the newborn’s biosignals (heart rate, heart rate variability, respiratory rate, SpO_2_, and systolic blood pressure). The power is wirelessly supplied through a large RF loop antenna transponder that allows a working distance of up to 25 cm, and real-time bio-signal data are transmitted to the user’s electronic device through BLE communication [37]. Figure 10a is a schematic of this module, where one EES mounted on the chest records the ECG data through a skin interface electrode composed of a fractal-shaped filament metal mesh microstructure, and the other attaches to the sole to record the PPG data. Figure 10b shows a schematic diagram of a device designed to perform the same function as a conventional wired device, powered by an antenna surrounding a user. Figure 10c compares the various biosignals measured by the ECG EES system and by the gold standard, which are almost identical.

As shown in previous studies, platforms that improve the discomfort of the existing system using the NFC method have practical applications in disease diagnosis and biosignal monitoring for patients sensitive to the external environment in hospitals. Research into NFC-enabled devices continues to target NICU infants as well as patients whose use of wired batteries or power supplies is limited due to severe skin trauma.

## 4. Conclusions

Conventional NFC technology has mainly been employed for user information identification and payment. However, research has been focused on wireless wearable devices that can non-invasively measure biosignals while making “skin-like” contact. The sensors that combine NFC and sensing technology presented herein measure various biosignals and have the objectives of system miniaturization and achieving wireless power supply and signal transmission. Optimized wireless power transmission efficiency is secured with a Q factor satisfied by ideal matching conditions through inductance control of the NFC coil antenna combined with individual sensor circuits. Furthermore, the low power consumption of the NFC chip ensures compatibility with single and multiple sensors, and circuit integration.

In this field of biosignals, the application of NFC technology is an excellent option to compensate for the shortcomings of conventional cumbersome and inconvenient wired devices. The systems for real-time monitoring of body temperature, pressure, electrophysiology, blood flow, sweating, etc., studied so far guarantee user convenience and respond immediately to biosignal abnormalities. Therefore, ultra-compact wireless wearable sensors combined with NFC technology are expected to become essential for biosignal analysis.
